# REPLY BY THE AUTHORS: Re: Persistent Mullerian Duct Syndrome: a rare entity with a rare presentation in need of multidisciplinary management

**DOI:** 10.1590/S1677-5538.IBJU.2017.0072.1

**Published:** 2017

**Authors:** Lin Da Aw, Murizah M. Zain, Sandro C. Esteves, Peter Humaidan

**Affiliations:** 1 Department of Obstetrics & Gynaecology, Hospital Sultanah Bahiyah, Kedah Darul Aman, Malaysia; 2 Fertility Clinic, Skive Regional Hospital, Denmark; 3 Androfert, Andrology & Human Reproduction Clinic, Referral Center for Male Reproduction, Campinas, Brazil; 4 Faculty of Health, Aarhus University, Denmark

*To the editor,*

Dr. de Jesus in his elegant commentary on our recent published article in the Int Braz J Urol (1) dwelled on the diagnosis challenges about patients with disorders of sexual differentiation (DSD) (2). Indeed, we appreciate and agree with his comments that given the rarity of this condition the differential diagnosis between Persistent Mullerian Duct Syndrome (PMDS) and other DSD, including mixed gonadal dysgenesis (MGD), represents a challenge for clinicians (3). In particular, these patients usually present for evaluation at later stages of sexual development, and a comprehensive clinical and laboratory evaluation is not always possible, as discussed below. Notably, as reasoned in our paper (2) and highlighted here, a multi-disciplinary team approach is essential to handle such conditions.

The diagnosis of PMSD in our patient was based on the presence of Mullerian duct derivatives - a fallopian tube, a bicornuate uterus with a more prominent right horn, and an enlarged cervix possibly hydrocolpos -with the presence of normal 46,XY karyotyping. The patient's mother provided the history of undescended testes with an ambiguity of genitalia, with no clear documentation, as the medical records are no longer available for perusal. However, there was no substantial evidence to support the ambiguity of genitalia. As for the authors’ remark that PMSD patients do not exhibit testicular failure, Claranette et al. suggested that such patients can be classified into three subgroups according to the position of reproductive organs: (i) Intra-abdominal Mullerian structures and testes in a position simulating that of the ovaries, (ii) one testis in a hernial sac or scrotum together with Mullerian, and (iii) both testes located in the same hernia sac along with the Fallopian tubes and uterus (4, 5). During laparoscopy, we identified two structures in the left pelvic region, one of which could represent an abdominal testis. We therefore believe the finding of a streak gonad might be compatible with the diagnosis of PMSD.

The typical features of PMDS include undescended testes and the presence of a small, underdeveloped uterus in an XY infant or adult, which both were found in our patient. This condition is usually caused by a deficiency of fetal anti-Mullerian hormone (AMH) effect due to mutations in the gene for AMH or the anti-Mullerian hormone receptor, however, may also be seen as a result of insensitivity to AMH of the target organ (6). Unfortunately, we were not able to investigate the AMH levels as the patient defaulted follow-up and treatment. The patient only recently sought treatment again for the problem of haematuria.

Although mixed gonadal dysgenesis (MGD) is similar in some ways to PMDS, the conditions can be distinguished histologically and by karyotyping. In our case, the chromosomal analysis was clearly 46,XY with no mosaicism. Unfortunately, a gonadal biopsy was not made as the diagnosis with karyotyping was thought to be sufficient in this case.
